# Monitor to innovate with feedback loops: process evaluation protocol for an anemia prevention intervention

**DOI:** 10.12688/gatesopenres.13417.2

**Published:** 2022-09-27

**Authors:** Ichhya Pant, Lipika Patro, Erica Sedlander, Shikha Chandrana, Rajiv Rimal

**Affiliations:** 1 Prevention and Community Health, George Washington University, Washington DC, USA; 2 IPE Global, Bhubaneshwar, Odisha, India; 3 Health, Behavior and Society, Johns Hopkins Bloomberg School of Public Health, Baltimore, USA

**Keywords:** neat to real time, monitoring, evaluation, feedback loops, social norms, rural, India, resource constrained

## Abstract

**Background: **Digital process monitoring and evaluation tools designed to capture near-to-real-time intervention data paired with feedback loops have the potential to innovate intervention delivery.

**Objective: **To describe how a multilevel social norms field trial (RANI) is using feedback loops to enhance intervention delivery.

**Methods: **We use a mixed-methods process evaluation design to monitor the Reduction of Anemia through Normative Innovations (RANI) project; a three-year randomized control trial which aims to lower rates of anemia among women in Odisha, India. Surveys and structured observation monitor fidelity to implementation and acceptability of implementation activities among study participants. Quantitative data evaluates implementation dose, coverage, exposure, and reach of intervention activities, and qualitative data will delve more deeply into reasons for high or low functioning. Iron folic acid supplement supply and demand are also monitored for stock-outs. Data collected from 130 intervention villages is processed, visualized, and triangulated in near to real-time via Real-time Monitoring for Knowledge Generation (RPM4K), a locally developed software application. Data visualization products facilitate the examination of monitoring data to mitigate bottlenecks and identify and implement tweaks to our intervention delivery strategy on an ongoing basis.

**Discussion:** Feedback loops facilitate timely course corrections. Feedback loops can also engender a shared understanding of ground realities for a geographically dispersed and culturally diverse team. Leveraging feedback loops, we identify opportunities to provide on-going supportive supervision for our community facilitators promoting joint problem-solving, and communication. Monthly media and hemoglobin level demonstration strategies are informed by participant engagement and acceptability. Stock-outs of iron folic acid tablets activate contingency plans to mobilize local stakeholders and advocate for timely resolutions. Unintended effects are monitored based on ongoing feedback from community facilitators.

**Conclusions: **Documenting our processes can inform the future implementation or scale up of similar projects embracing feedback loops to iterate and innovate their intervention delivery.

## Introduction

### Anemia endemic among women of reproductive age

Anemia affects roughly a third of the world's population, making it a major global public health problem that impacts maternal and child mortality, cognitive and physical performance ^1^, and work capacity and productivity ^2, 3^. In India, more than 50% of women of reproductive age have anemia ^3^. The largest contributor to anemia worldwide is iron deficiency ^4^. Despite concentrated efforts and the presence of several government programs (e.g., National Iron Plus Initiative and Anemia Mukt Bharat) ^5^, which offer women of reproductive age free iron supplements ^6– 9^, anemia prevalence remains high ^3^. India’s Demographic Health Data (2015–16) shows that only 30% of pregnant women consumed iron folic acid for more than 100 days during pregnancy ^3^.

Several studies have examined supply side issues to IFA consumption ^10– 13^, but studies on demand side challenges including knowledge and social barriers are limited ^14^. Recently, researchers have begun to ask questions about the role that community-level factors, such as social and gender norms, can play in women’s IFA consumption ^15– 18^. The Reduction in Anemia Through Normative Innovations (RANI) project seeks to bridge this gap with a multilevel intervention focused on increasing demand for IFA by influencing anemia-related social norms ^19^.

### The RANI (Reduction in Anemia through Normative Innovations) project

The RANI project is a cluster randomized trial launched in 2019 in two blocks, Athamalik and Kishorenagar, within the Angul district of Odisha on the eastern coast of India. These blocks are spread over 1278 sq. kilometers and they constitute a total of 558 villages with a total of 218, 373 people in greater than 50,000 households per the 2011 census ^20^. Here, the majority of residents (83%) live in rural areas, a third are literate, and primarily identify as Hindu ^20^. Like the rest of the country and the district of Odisha, India, almost half of all women of reproductive age are anemic ^14^. In the study, there are 89 selected clusters of villages, which we randomized into treatment and control on a 1:1 basis. The usual-care control arm receives no intervention, whereas the treatment arm receives the RANI project components. In total, 15 clusters (40–41 villages) were selected and 4000 women (2000 in each arm) living in the selected clusters were randomly selected to take part in data collection.

**Figure 1.**
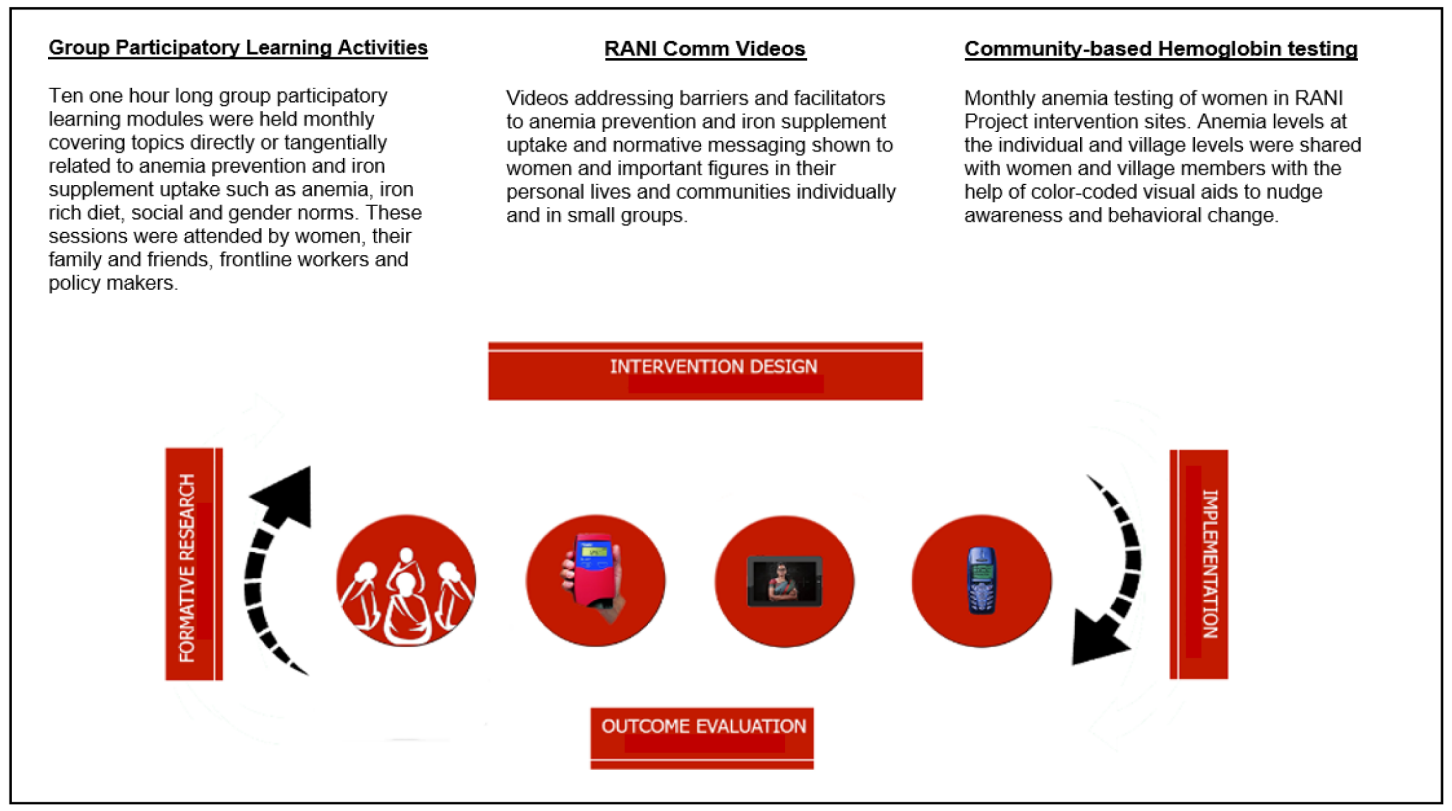
Visualization and description of RANI Project intervention components.

The RANI Project’s overall implementation approach, based on its goal to improve IFA consumption through a social norms-based intervention, includes a number of intervention components (see Figure 1 for a visual description of each intervention component), which require monitoring and iterating the intervention delivery strategy at multiple ecological levels throughout the implementation period. Ethical approvals to conduct the RANI project have been acquired from appropriate institutions within the United States and in India (see Yilma *et al*. 2020 for the full intervention protocol ^19^).

### Process evaluation framework

In public health interventions, process evaluation allows researchers and practitioners to understand what works, what does not work, for whom, and under what conditions. It also serves the important function of providing ongoing feedback so that interventions can be responsive to the changing conditions in the field ^21– 23^. Process evaluations also help determine whether fidelity to the implementation protocol was maintained during the course of the intervention period, whether intended participants were reached, and if any unintended consequences resulted from the project ^24, 25^.

Health care delivery and outcomes can be improved by using innovations (i.e., new ideas, technologies, and practices) supported by scientific evidence. Over the last couple of decades, process evaluation methodologies have been evolving ^21^. They were originally centered on qualitative research alongside trials and were conducted to provide a deeper understanding of the disease condition, implementation issues and mechanisms of the intervention ^22^. Now, there is growing consensus that qualitative and quantitative data (mixed methods) can help facilitate trial implementation, identification of the effectiveness of “active ingredients”, and research translation. As methods have advanced, integration of feedback loops is emerging as the next methodological frontier for process evaluations.

### Use of feedback loops in Process Monitoring and Evaluation

Feedback loops are mechanisms that allow intervention information to flow back to the program implementers on an ongoing basis so that appropriate changes in intervention delivery strategy can be adopted throughout the implementation period. Incorporating feedback loops into the process evaluation design ensures interventions are meaningfully and strategically responsive to emerging requirements. In modern times, these loops increasingly rely on mobile and other forms of digital technology, which improves accuracy and reduces the time lag between events on the ground and program adaptations. As such, feedback loops can improve decision-making throughout the intervention.

 Historically, institutionalizing feedback loops as an integral part of process evaluations has proven to be challenging ^26– 29^. Many scholars ^29^ have characterized the need for feedback loops “as a bold challenge to current orthodoxy aided by developments in theory, methods, and practice” (p. 3). They call for an approach that promotes interaction between project designers, implementers, researchers, and decision-makers to encourage adaptation through learning. Such an approach would center on agility, responsiveness, a culture of experimentation, responsiveness, and relevant, timely, actionable data as its key characteristics. Furthermore, scholars advocate moving away from current dominant models of static intervention design and implementation, which tend to be modeled after trials that do not permit projects to be responsive to the complexities and unpredictability of implementation challenges that arise in social and behavioral intervention trials ^29^.

The use of feedback loops is, however, mainstream in the commercial sector for corporations such as Uber, eBay, and Airbnb ^30– 33^. Similarly, there are several examples of improved implementation delivery and outcomes by leveraging feedback loops. They range from engaging millions of youths in informing policy, improving use of evidence in decision-making, improving flood response time, and efficiency in HIV treatment targeting in Zimbabwe ^34– 40^. Citizen reports of drug stock-outs and improvement in community health worker performance ^34, 41– 43^ are other examples of the effective application of feedback loops ^34, 44– 46^.

### Motivation for incorporating feedback loops

The synergy between feedback loops integrated within process evaluations and enhancement of implementation delivery is still an under-explored area, especially in the context of randomized control trials (RCTs) ^41^. One of the monitoring goals of the RANI Project was to reduce this knowledge and implementation gap. Specifically, we describe the process evaluation methods we are adopting in a multilevel intervention to increase iron folic acid supplement use for anemia prevention in Odisha, India.

In sum, the synergy between feedback loops integrated within process evaluations and enhancement of implementation delivery is still an under-explored area, especially in the context of randomized control trials (RCTs) ^47^. One of the monitoring goals of the RANI project was to reduce this knowledge and implementation gap. Specifically, we describe the process evaluation methods we are adopting in a multilevel intervention to increase iron folic acid supplement use for anemia prevention in Odisha, India.

## Methods

We use a mixed methods approach which has largely benefited from key pieces of literature ^35, 47, 48^ that detail systematic approaches to designing and incorporating feedback loops to facilitate adaptive management for health improvement interventions, and we have adapted them on an ongoing basis to meet the RANI project’s specific needs. Quantitative data is collected via surveys and structured observation is used to monitor implementation fidelity (i.e., dose, coverage, exposure, reach of intervention activities, village-level supply of and demand for iron folic acid supplements), and acceptability among program participants. In parallel, monthly qualitative reports provide insights into barriers and facilitators to intervention delivery. Overall, our globally dispersed team leverages near to real-time data to mitigate bottlenecks and pain-points as well as leverage opportunities to enhance implementation delivery. We collectively identify, agree upon, and incorporate tweaks to our intervention delivery strategies on an ongoing basis.

### Ethics approval and consent to participate

Approval to conduct the study was gained from The Institutional Review Board at The George Washington University (FWA00005945) and the Sigma Science and Research, an IRB located in New Delhi, India (10031/IRB/D/18-19). This trial was registered with Clinical Trial Registry- India (CTRI) (CTRI/2018/10/016186) on 29 October 2018. All participants went through a verbal and written informed consent process before data collection.

### Process evaluation aims and questions

The RANI project process evaluation has three aims.

1. To assess fidelity to implementation while guiding intervention planning and delivery for the larger trial via feedback loops.
2. To monitor the quality of intervention delivery and acceptability of the intervention among its intended audiences.
3. To monitor the supply and demand of iron folic acid supplement in intervention delivery sites to mitigate possible supply chain disruption.

Our process evaluation questions are anchored in the RANI project’s process evaluation framework and aims. They were finalized in collaboration with our stakeholders ( Table 1), who serve as key members of the feedback loop decision-making sub-team (i.e., principal investigator, project director, M&E (monitoring and evaluation) technical lead, intervention implementation and evaluation manager) within our project.

**Table 1.** Process evaluation questions.

1. To what extent was fidelity to implementation maintained during the implementation of the T4 sessions, community engagement meetings, media demonstrations, and hemoglobin testing sessions and demonstrations? a. What were the total number of implementation sessions conducted per village? b. Were community facilitators adequately supported during implementation delivery? c. Were T4 sessions informative and interactive for our program participants? d. What was the level of exposure to our media products at the village-level? e. What were our program participants’ reactions and acceptability of our implementation activities? f. What were the barriers and facilitators faced by our community facilitators and our target population? g. Were there any potential unintended consequences (neutral, positive or negative) as a result of our program? h. Was there adequate supply of Iron Folic Acid supplement at the village level? i. Did demand for Iron Folic Acid supplement increase or decrease at the village-level during the implementation period?
2. Did we reach RANI’s target audience with our implementation activities? a. What was the total reach of our implementation activities? b. Who did we reach with our implementation activities?

Following the finalization of our evaluation aims and questions, we developed a logic model and M&E planning framework (See Online Repository). This framework details key inputs, outputs, outcomes (short, intermediate, and long-term) and links them with data collection instruments. It outlines the frequency of data entry, responsible parties, reporting frequencies, data visualization tools, and a plan for disseminating our findings. Furthermore, the framework served as a guiding document when developing the wireframe and final design for our process evaluation software application, called the Real-time Performance Monitoring for Knowledge (RPM4K).

### Real-Time Performance Monitoring for Knowledge (RPM4K)

**Figure 2.**
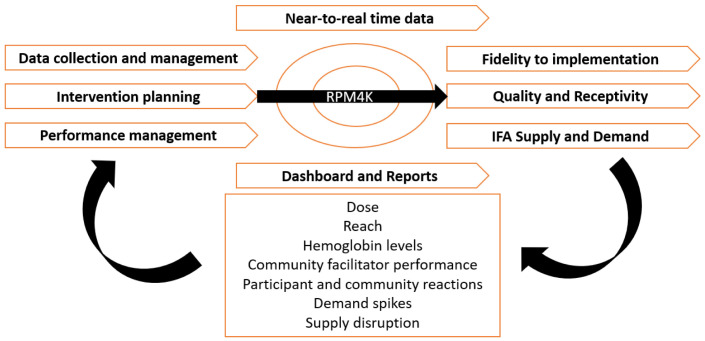
RANI Project’s Process Evaluation Conceptual Framework.

RPM4K is a software application customized and developed locally for the RANI Project process evaluation. It facilitates intervention planning, delivery, performance monitoring, and automated production of data visualization and reports using near to real-time data to identify and inform feedback loops ( Figure 2). RPM4K application offers a dual mobile application and a web-based interface.

The process evaluation data lifecycle centers and revolves around our community facilitators and their cluster supervisors. RANI community facilitators, who serve as frontline workers to deliver intervention components, use the system to enter field level data with the help of a hand-held (mobile) device. Their cluster supervisors use the mobile as well as the web interface for entering supervision data and also monitoring intervention delivery and quality by reviewing data entered by RANI community facilitators. Key stakeholders use the web interface to monitor the pace and delivery of intervention activities of both cluster supervisors and facilitators and utilize aggregated data to identify feedback loops and implement tweaks to the intervention. Access to RPM4K is tiered at multiple levels.

Community facilitators are only able to access their own performance data as well as intervention data for the villages they work in. Similarly, their cluster supervisors can only access performance data for the community facilitators they manage and the villages they work in. Key members of the process evaluation team (e.g., process evaluation lead, state implementation manager, project managers etc.) are able to access and view intervention data for all villages and performance data for all personnel. However, the system administrator is the only person with access to all structural functionalities of RPM4K. They are responsible for mapping intervention villages to cluster supervisors or community facilitators and defining data access for all users.

At the beginning of each month, the system administrator is responsible for uploading intervention plans onto RPM4K. They direct the RANI project’s process evaluation data lifecycle, quality control, and technical assistance requests from RPM4K end users. At month’s end, data on key performance indicators is auto aggregated into dashboards and reports in the form of easy to interpret heat maps and visual reports. Raw data for specific time periods or the entirety of the intervention delivery period can also be downloaded from RPM4K by users with defined data privileges.

### Data collection

We collect, process, visualize, and triangulate quantitative, qualitative, and administrative data from 130 intervention villages in near to real-time using RPM4K. Data collected ranges from the individual level (e.g., community facilitator performance and hemoglobin levels) to the village-level (e.g., IFA supplement supply and demand). Key performance indicators for each ecological level are summarized in Table 2. A data quality assurance protocol is in place to review various levels and layers of data in a collaborative manner (See Supplementary Material). Data entry, management, review occurred in monthly cycles. Frontline community facilitators enter data in near-to-real time. Their supervisors conduct the first round of data review followed by a final check of data quality by the Data Manager prior to approving submitted data. Data quality review focused on conducting range checks for data values, monitoring and removing duplicate, blank or outlier values, timeliness with encoded deadlines for data entry submissions, completeness check against monthly implementation plans. All data was housed in a secure cloud server with full privileges limited to the Data Manager only.

**Table 2.** Key performance indicators summarized at various ecological levels.

MEASURE	INDICATORS	Individual	Interpersonal	Village
1. Fidelity – Dose	1.1 Number of participatory learning acitivity sessions			**X**
	1.2 Number of media demonstrations			**X**
	1.3 Number of women participating in Hemoglobin testing	**X**	**X**	**X**
	1.4 Number of community engagement meetings		**X**	**X**
	1.5 Number of hemoglobin testing demonstrations	**X**	**X**	**X**
	1.6 Number of women tested as anemic (mild, moderate, or severe)	**X**		**X**
	1.7 Number of referrals to community clinical linkages for anemic women	**X**		**X**
	1.8 % of self-reported iron folic acid supplementation intake status (currently taking, previously taken, or never taken)	**X**		**X**
2. Quality – Reach	2.1 Number of women of reproductive age reached			**X**
	2.2 Number of adolescent girls reached			**X**
	2.3 Number of mothers-in-law reached			**X**
	2.4 Number of pregnant women reached			**X**
	2.5 Number of frontline workers reached			**X**
	2.6 Number of policy workers reached			**X**
	2.7 Number of men reached			**X**
	3.1 CF performance	**X**		**X**
	3.2 Qualitative barriers, facilitators, challenges, or opportunities	**X**	**X**	**X**
	3.3 Unintended consequences (neutral, positive or negative)	**X**	**X**	**X**
4. Acceptability – Reactions	4.1 Number of thumbs up for comm products			**X**
	4.2 Number of thumbs down for products			**X**
	4.3 Number of thumbs up for participatory learning acitivitysessions			**X**
	4.4 Number of thumbs down for participatory learning acitivity sessions			**X**
	4.5 Number of attendees willing to return to participatory learning acitivity sessions and community engagement meetings			**X**
	4.6 Number of returning attendees for participatory learning acitivity sessions and community engagement meetings			**X**
	4.7 Qualitative barriers, facilitators, challenges, or opportunities	**X**	**X**	**X**
	4.8 Unintended consequences (neutral, positive or negative)	**X**	**X**	**X**
5. Iron Folic Acid – Supply and Demand	5.1 Number of iron folic acid supply points with readily available stock			**X**
	5.2 Number of iron folic acid supply points with IFA in short supply			**X**
	5.3 Number of iron folic acid supply points with IFA stock-outs			**X**
	5.4 Number of iron folic acid supply points reporting an increase in demand			**X**
	5.5 Qualitative barriers, facilitators, challenges, or opportunities	**X**	**X**	**X**
	5.6 Unintended consequences (neutral, positive or negative)	**X**	**X**	**X**

### Monthly qualitative summary report

RANI project community facilitators submit a monthly qualitative report summarizing their reflections, perceptions, and experiences regarding the quality of their intervention sessions, self-assessment of their performance, participant and community reactions and acceptability to intervention activities, barriers and facilitators and unintended consequences, if any (See Online Repository for the full interview guide). All open-ended responses get captured in RPM4K in Odiya, the local language, and then get translated and deductively summarized by the Data Manager using the interview guide to formulate themes in a monthly report written in English using Microsoft Word.

### Quantitative measures and instruments

The majority of quantitative data is collected and entered into RPM4K through the Community Facilitators Input Form (submitted by community facilitators), the Community Facilitators Evaluation Form (submitted by their cluster supervisors). These forms have dedicated sections dealing with key performance indicators for each intervention component (i.e., T4, Hemoglobin testing, RANI Comms, IFA supply and demand status, etc.). The forms also have built-in global positioning system (GPS) and activity image capture features to associate location and visual data with intervention activities. All forms are submitted within four days of completing an intervention activity. See Online Repository to review our data collection instruments and screenshots of RPM4K interface, dashboards, and data visualization products.

### Dose

Dose is operationalized as the total number of intervention activities (e.g., T4 sessions, community engagement meetings, RANI Comm video demonstration sessions, hemoglobin testing sessions and results demonstrations) reported on RPM4K and summarized at the village-level by algorithms embedded within RPM4K.

### Reach

Reach is measured as the total number of women of reproductive age, their families (i.e., husbands and mothers-in-law), frontline workers and policy makers in the village exposed to intervention activities. Reach is summarized at the village-level by algorithms embedded within RPM4K.

### Acceptability

Acceptibility of intervention components is operationalized as the total number of likes versus dislikes shared by program participants following the delivery of an intervention activity by RANI community facilitators. Acceptability is aggregated at the village-level by algorithms embedded within RPM4K as the total number of likes and dislikes allocated to all delivered intervention components. Acceptibility is also assessed via qualitative feedback noted by RANI community facilitators made on a monthly basis ( *see* supplementary materials to review how we captured acceptability of RANI intervention components via the monthly generated qualitative reports). 

### Community facilitator performance

Community facilitator performance is assessed and monitored using three distinct tools implemented at different timepoints. The first tool measures the baseline professional abilities of RANI community facilitators when they are first onboarded as part of the RANI project implementation team. Each RANI community facilitator is classified on a scale of 1 (exceptional) to 3 (needs improvement) points on six measures – 1) their prior work experience in participatory learning activities or community mobilization, 2) communication skills, 3) professional network, 4) familiarity with the intervention area, 5) references from prior colleagues or supervisors, 6) logistical preparedness related to job requirements.

The second tool for assessing and monitoring community facilitator performance is integrated within RPM4K. It consists of a self-assessment scale reported by community facilitators following every intervention session followed by monthly cluster supervisor assessment of community facilitators. The community facilitator self-assessment scale consists of three measures which ask 1) how easy or difficult it was to deliver the intervention session they’re reporting on, 2) how much the community facilitator thinks participants understood each session, and 3) how they would rate their overall performance for the session they just delivered. The response options consist of five-point Likert scales ranging from very difficult to very easy, did not understand at all to understood all of the content, and very poor to very good, respectively. Similarly, cluster supervisors conduct random visits covering at least 10% of all intervention sessions delivered by a community facilitator during any given month. They perform intervention session evaluations and report community facilitator performance by observing and assessing their pace, accuracy, confidence, and ability to hold the audience’s attention when delivering intervention components. The response options consist of five-point Likert scales ranging from too slow to too fast and inaccurate to accurate; three-point Likert scales ranging from not at all confident to very confident and not at all focused to very focused. Data from these two tools are summarized at the village-level by algorithms embedded within RPM4K.

The third and final tool is utilized and reported with varying periodicity ranging from monthly to quarterly assessments while community facilitators work in the field. Each RANI community facilitator is classified on a scale of 1 (exceptional) to 3 (needs improvement) points on several measures, which assess programmatic and human resource related performance such as a community facilitators’ ability to deliver intervention content and respond to intervention feedback loops in a timely manner, need for supportive supervision, and community mobilization skills. This data is collected by the district implementation management team and analyzed using Microsoft Excel.

### IFA supply and demand

In 2019, the RANI project’s implementation leadership team based in Bhubaneshwar, Odisha conducted an initial assessment to map the IFA supply chain, supply status and monitoring techniques in intervention villages before intervention delivery began. Using this information, they operationalized how to monitor IFA supply status, developed tentative ideas for streamlining IFA supply in the intervention villages, and designed an IFA supply and demand monitoring module for RPM4K. IFA supply status is measured as the total number of supply points reporting adequate supply, shortage of supply or stock-outs. Similarly, IFA demand is measured as the total number of supply points reporting an increase in demand. IFA supply and demand is summarized at the village-level by algorithms embedded within RPM4K.

### Hemoglobin levels

Hemoglobin testing gets conducted monthly among 15 women, using a HemoC ue photometer (model HB 301), which provides instant results in terms of hemoglobin concentration in gm/dL (grams per deciliter). This group consists of five women who volunteered as repeat testers every month and ten women who have not been tested before through the RANI project. Test results are shared as color coded (green – anemia free; yellow – mild anemia; orange – moderate anemia; red – severe anemia) blood shaped cards visualizing severity of anemia or lack thereof. Post testing, recommendations for behavioral actions and nudges are tailored according to participant’s test results. Measurement and monitoring of anemia levels at the village-level are analyzed and visualized using embedded algorithms and heat maps on RPM4K subsequently to monitor trends at the village-level.

### Identifying, documenting and implementing feedback loops

Generally, process evaluation data are collected and analyzed at the end of the intervention delivery period, which limits the understanding and insights stakeholders have on barriers and facilitators on the ground ^8^. The merger of feedback loops with applications such as RPM4K allow complex, multi-component interventions like RANI to assess ground-realities to innovate intervention delivery strategies in near-to-real-time by triangulating quantitative intervention process monitoring data, qualitative monthly reports, and administrative data related to community faciliator performance. To do this systematically and efficiently, for every scenario that emerges, the M&E team first triangulates quantitative and qualitative monitoring data collected on a near-to-real-time basis and then identifies relevant tailoring variables ^48^. They are defined as variables that serve as indicators of when an intervention delivery strategy requires optimization or calibration by the intervention delivery team. For the RANI project, the optimization or calibration takes the form of feedback loops. Table 3 provides several examples of hypothetical scenarios, measures, tailoring variables, and potential feedback loops identified to explicate this process in practice. All feedback loops are currently documented in an offline tool and will be built on as a formal module of RPM4K during future upgrades to the application (see Online Repository to review the RANI Project’s Feedback Loops Documentation Tool).

**Table 3.** Hypothetical Examples of Tailoring Variables and Feedback Loops.

SCENARIO	MEASURES	TAILORING VARIABLE	FEEDBACK LOOPS
There are waitlists to get Hemoglobin testing by women in X % of villages or X village the week the intervention kicked off	Fidelity – Dose	Hemoglobin testing resource allocation	○ Continue to monitor demand (i.e., number of villages with waitlists and number of women on waitlists) ○ Refer women to the near clinical services where they can get tested for anemia ○ Pivot intervention resources towards allocating more resources towards testing
Community facilitator performance is low for X % of Community facilitators or Community facilitators from X % of supervisory teams	Quality – Community facilitator performance	Supportive supervision	○ Identify right type and time of support needed for community facilitator ○ Identify right type and time of support needed for community facilitator
Iron folic acid supply is low X% of villages	Iron folic acid supply	Policy advocacy	○ Continue to monitor IFA stock-outs ○ Engage local officials and frontline workers to advocate for iron folic acid supply in impacted villages ○ Refer women to private supply points where iron folic acid supply is readily available although may not be free of cost ○ Refer women to nearby villages where iron folic acid is readily available
Rumors regarding Activpal utilized during baseline data collection by another firm	Unintended Consequences	Rumor management	○ Designate as urgent or non-urgent matter ○ Continue to monitor prevalence of rumors ○ Shed light and awareness on the instrument

Tailoring variables for the RANI Project primarily focus on:

**1] Supportive supervision to bolster community facilitator performance.** In general, a group of 7-8 RANI community facilitators are supported by a cluster supervisor. Overall, the supervisor helps the community facilitator gain mastery over the session content by organizing field level demonstrations. The supervisor provides on-site support in mobilizing participants and delivering intervention sessions. They provide constructive feedback to the community facilitator for improving session quality and participation levels based on both direct observation of session delivery and interaction with community members. Any discussion with policy makers and sections of the village which are unwilling to participate in the intervention or coordination with local health care officials are also facilitated with support from cluster supervisors.

Beyond cluster supervisors, the intervention management team also conducts monitoring visits and provides feedback to the field teams for improving intervention delivery. All broader issues identified in the field are shared during monthly meetings. Protocols and audio recordings for improving intervention delivery in local vernacular are developed for community facilitators and cluster supervisors on a regular basis. The entire intervention delivery team is also part of WhatsApp groups where updates, plans, requests for technical assistance for day-to-day activities are shared by all team members with instant feedback by the intervention management team.

**2] Refining RANI comm strategies**. We assess how each video resonates with our program participants informed by acceptability indicators (both qualitative and quantitative) for RANI comm videos data on RPM4K. For example, if we find that adolescents are more receptive to the videos than older women, we pivot to show videos at events where adolescents are more likely to attend (e.g., a festival or school-related event) and increase other intervention activities among older women.

**3] Altering hemoglobin testing strategies.** We alter our implementation plans based on hemoglobin testing data. For example, when we find that there is a high demand for hemoglobin testing in our intervention villages, we review quantitative and qualitative reports to examine how test results are being perceived and if they are influencing IFA use. If the reports indicate that hemoglobin testing is having the desired effect, we consider increasing the number of women that we test while remaining mindful of resource constraints.

**4] Policy advocacy to accommodate IFA supply or demand disruption.** In the Angul district of Odisha, the government provides daily IFA supplementation for pregnant women and weekly IFA for adolescents for free. While adolescents and pregnant women can acquire IFA free of cost, non-pregnant women of reproductive age remain uncovered. Keeping this coverage gap in mind, the RANI project intervention team assesses and shares requirements for providing IFA supplements to non-pregnant women with local officials and frontline workers on a monthly basis. Every month after village-level hemoglobin testing is completed, aggregated test results are shared with local health care workers and higher-level administration at the intervention sites. This information is supplemented with a demand for IFA as per the aggregated anemia levels of pregnant and non-pregnant women of reproductive age in the intervention villages. IFA stock is usually replenished and delivered within a fortnight. In case of any delay or shortfall, the issue is escalated first at the block level and then to the district level, if required.

**5] Engaging in participatory consensus building with all stakeholders.** The decision-making process we’ve adopted is participatory and based on 360-degree feedback from all key stakeholders. The intervention plan for a month is developed during monthly meetings of the intervention team with feedback incorporated from the process evaluation sub-team prior to seeking feedback from our intended program participants and local stakeholders. Next, the village-specific intervention plans are shared with women of reproductive age in our intervention villages, members of local influential groups, policy makers (sarpanch, ward members etc.) and frontline workers before finalization. Once the feedback loops are implemented, RANI community facilitators solicit comments from the participants on the session content and facilitation quality. Comments are also sought by supervisors and the intervention management team during monitoring visits via informal opinion polls. The quantitative ratings are recorded in RPM4K’s mobile-based forms, whereas qualitative feedback is compiled in monthly qualitative reports shared with all stakeholders. Field-level findings, intervention achievements and qualitative findings are shared with the process evaluation team on a weekly basis. These discussions, along with interaction with the field teams, is used to tweak RANI’s intervention delivery strategy on a monthly basis.

Monthly coordination and advocacy meetings are also conducted with block and district level government counterparts from the Departments of Health, Women and Child and Odisha Livelihood Mission. Village-wise hemoglobin status along with demand for IFA tablets is discussed during these interactions. The district collector (local government leadership) holds quarterly review meetings with all officials from the local departments involved with RANI. Through these interactions, local government officials also provide feedback and suggestions for improving the coordination and convergence of the RANI project with other ongoing anemia and health improvement programs in the district.

### Survey data triangulation and analysis

Beyond using feedback loops to inform RANI intervention tweaks, we plan to triangulate RANI’s endline survey data with our monitoring data to assess whether intervention delivery factors (such as village-level reach, community facilitator performance over time, testing coverage, implementation coverage, IFA supply and demand) influence intervention exposure levels at the village-level. We will also explore whether and how 1) these intervention delivery factors influence psychosocial (e.g., self-efficacy, knowledge, awareness, risk perception), normative or behavioral (e.g., IFA use and adherence) outcomes at the village-level controlling for village-level demographics (i.e., age, income, education, caste) and structural factors (e.g., proximity to supply points). Finally, we will assess whether intervention exposure at the village-level mediates the relationship between village-level intervention delivery factors and psychosocial, normative, or behavioral outcomes.

## Discussion

The purpose of this paper is to shed light on the RPM4K, a process evaluation and monitoring system being implemented by the RANI Project (which has now completed its implementation delivery with final impact evaluation underway). It is built on the idea that even the best designed interventions are not able to foresee all changes that occur as project activities are rolled out. The overall environment is likely to change (as was the case in this study with the advent of coronavirus disease 2019 (COVID-19) that required significant delays and adaptations) as are the social and political realities on the ground. Furthermore, changes brought about by the intervention can themselves precipitate other disturbances in the system – as was the case when our campaign to generate demand for IFA resulted in shortages in supply.

Given these realities, and many others that cannot be anticipated at the beginning of the intervention, it is critical to build feedback loops into the overall implementation plan so that changes can be incorporated on an ongoing basis. This requires at least three criteria to be met. First, the different sequential steps in data processing (collecting, analyzing, and reporting) have to occur with minimal delays. In the RANI Project, this meant data collection in real (or near to real) time, automated algorithms built into the analysis step, and a designated person for analysis and reporting on a predetermined timeline.

Second, adaptations made to the intervention rollout plans because of monitoring feedback have to be visible to the team. When data collectors and others involved in data processing come to view their efforts as being inconsequential (because adaptations are not being made to the intervention based on feedback received), few incentives remain for them to continue their work with full commitment. Conversely, the ability to see changes in the intervention as a result of one’s data collection, processing, or reporting activity can serve as powerful motivators to continue and even improve one’s performance. The RANI project held monthly meetings with facilitators, supervisors, and RANI’s state implementation manager during which time changes made to the implementation schedule and activities were shared with the team. More so, we’ve engaged in community and policy-level dissemination events on a monthly basis informing our stakeholders and intervention recipients of changes in social norms, anemia as well as iron folic acid supply, demand, and uptake within their localities. Disseminating the knowledge we’ve gathered from our monitoring and survey data has been an integral part of feedback loops built into the RANI intervention delivery strategy. Beyond our local stakeholders and intervention recipients, we’ve engaged in dissemination via regional, national and international conferences, webinars, academic journals, social media platforms, and media outlets.

Finally, it is also important that the overall team buy into a culture of innovation. This is likely the most difficult task, particularly for projects perceived as being conceptualized and implemented externally, with minimal local stakeholder input, and ones thought to be driven solely by outcomes, without regard for underlying processes. The idea that a project is open to innovations means that failures are anticipated, allowed to happen without retribution, and framed in terms of learning opportunities for approaching the task in a way different from how it was originally done. The process evaluation protocol for the RANI project, detailed in this paper, provides a blueprint for other programs or trials aiming to harmonize responsive feedback loops, (near to) real-time data, and adaptive tailoring variables to enhance their intervention delivery and overall impact.

## Conclusion

The process of incorporating of feedback loops paired with digital intervention tools which offer near-to-real-time intervention data remains a gap in literature despite calls from leading experts in the field. This protocol bridges this gap and contributes methods, tools, templates, instruments, processes, frameworks, and a publicly available software that facilitate the incorporation of feedback loops to innovate the intervention delivery of the RANI project.

### Study status

The RANI study was implemented between September 2018 and March 2021.

## List of abbreviations

**IFA**- Iron and Folic Acid

**RANI**- Reduction in Anemia Through Normative Innovations

**USAID**- The United States Agency for International Development

**RCTs**- Randomized Control Trials

**M&E**- Monitoring and Evaluation

**RPM4K**- Real-time Performance Monitoring for Knowledge

## Data Availability

Figshare. The RANI Project Process Monitoring and Evaluation Dataset and Codebook. DOI: https://doi.org/10.6084/m9.figshare.16709452.v1 ^49^. This project contains the following underlying data: RANI Project Process Monitoring and Evaluation Dataset RANI Project Process Monitoring and Evaluation Data Dictionary Figshare. RPM4K Supplementary Files. DOI: 10.6084/m9.figshare.20186837 This project contains the following extended data: Appendix 1: Sample Template for RANI’s Monitoring and Evaluation Framework Appendix 2: Qualitative Interview Guide Appendix 3: RANI M&E Sample Data Collection Form Appendix 4: RPM4K Dashboard and Data Visualization Tools Appendix 5: Feedback Loops Documentation Tool Appendix 6: RPM4K Data Quality Protocol Data are available under the terms of the Creative Commons Zero "No rights reserved" data waiver (CC BY 4.0 Public domain dedication).

## Software availability

Zenodo. ipant/RANI: RANI DOI. DOI: https://doi.org/10.5281/zenodo.5062358 ^50^

Data are available under the terms of the Creative Commons Zero "No rights reserved" data waiver (CC BY 4.0 Public domain dedication).
