# Supplementary material for: Monitor to innovate with feedback loops: process evaluation protocol for an anemia prevention intervention
Source: Gates Open Res. 2022 Sep 27;6:13. Originally published 2022 Mar 16. [Version 2] doi: 10.12688/gatesopenres.13417.2 (PMC9525569; doi:10.12688/gatesopenres.13417.2)
Supplement: Peer review history

# Peer review: Reviewer response for version 2

**DOI:** 10.21956/gatesopenres.15310.r32614

**Published:** 2022-09-30

**Authors:** Ankit Anand

**Affiliations:** 1 Population Research Centre, Institute for Social and Economic Change, Bangalore, Karnataka, India

The problem here is that the all of the intervention is already published and the trial have also been approved. This paper is an extension on the monitoring part of the intervention. It gives a very little scope to review this as an original contribution. Additionally, it will be helpful to read with the actual intervention.

 In that case, I would recommend indexing.

Is the study design appropriate for the research question?

Yes

Is the rationale for, and objectives of, the study clearly described?

Partly

Are sufficient details of the methods provided to allow replication by others?

No

Are the datasets clearly presented in a useable and accessible format?

Yes

Reviewer Expertise:

Maternal and Child Health, Adolescent Health, mHealth, Health System, Evaluation Framework

I confirm that I have read this submission and believe that I have an appropriate level of expertise to confirm that it is of an acceptable scientific standard.

# Peer review: Reviewer response for version 1

**DOI:** 10.21956/gatesopenres.14672.r32055

**Published:** 2022-05-27

**Authors:** Ankit Anand

**Affiliations:** 1 Population Research Centre, Institute for Social and Economic Change, Bangalore, Karnataka, India

The study describes the RANI project extensively. The protocol provides good detail and indicators for monitoring and evaluating the project. However, I feel it needs a lot of improvement in its current form

- The authors need to include details about the geography and the motivation for this project. The process of arriving at this intervention is somewhat mentioned in different sections but needs to be more clear in the introduction part.
- The protocol states using an innovative digital tool as an intervention. I would think the videos are innovative tools. However, the program focuses on multiple actors/aspects of the communities and even intervenes on the demand side of the issues. I think it is a quite comprehensive intervention but could not understand the innovation part of it.
- How will the innovation be linked to improvement in outcome, for example in measuring dosage? Almost all of the activities (sessions, video demonstration sessions, haemoglobin testing sessions, and results demonstrations) are combined and considered as dose. It will be impossible to point out which activities are more effective, or which need more improvements.
- The same thing for receptivity, I could not understand how likes versus dislikes will measure receptivity? More nuances of receptivity need to be there.
- The intervention, especially the videos seem to be open-ended intervention which could be called innovation? Feedback loops are usually part of most digital intervention strategies.
- However, I really appreciate the comprehensiveness of the approaches which may lead to a better outcome but evaluating activities and attributing effectiveness to all these activities will be a challenge. It might be related to more efficiency in their fieldwork/community engagements activity rather than the innovation. Which one (algorithms or procedures) leads to changes?
- The replicability of the intervention will also be difficult.
- More clarity needed on sources of data. Who will be interviewed? What information will be collected from each set of respondents and how it will correspond to the aims of the project? It is somewhat mentioned in table 2 but not able to know how it will be measured.
- Too many evaluation questions? For example: "level of exposure to our media products" is an intermediate or process outcome, it can not be a final outcome.
- A diagrammatic version of the theory of change might help in understanding.
- The protocol mentioned has three very different aims. What are the areas the authors see as a problem? Whether it is a supply-chain, human resources, community engagements or demand-side issue? That is why I feel a paragraph stating the geography and their challenges is needed. It also needs to be linked with the motivation of the intervention. The first part of the introduction is very generic and does not link to the second part. The authors needs to provide specific details not generic statements.
- A clear timeline for the intervention and process of feedback loops must also be clearly mentioned. If not then the intervention becomes non-replicable.

Is the study design appropriate for the research question?

Yes

Is the rationale for, and objectives of, the study clearly described?

Partly

Are sufficient details of the methods provided to allow replication by others?

No

Are the datasets clearly presented in a useable and accessible format?

Yes

Reviewer Expertise:

Maternal and Child Health, Adolescent Health, mHealth, Health System, Evaluation Framework

I confirm that I have read this submission and believe that I have an appropriate level of expertise to confirm that it is of an acceptable scientific standard, however I have significant reservations, as outlined above.

# Response

**Published:** 2022-09-18

**Authors:** Ichhya Pant

**Affiliations:** GWU School of Public Health, USA

Thank you for reviewing our manuscript and offering your feedback. We offer the following point by point response to address the concerns raised by you in your review.

 It may be that the reviewer is asking about the motivation for the intervention, given the geographical area where the work is done. We note, however, that this paper is about the monitoring of the intervention and not the intervention itself (for which we have already published quite a bit, including provide a link to Yilma *et al.* and Sedlander *et al.*)  But we have taken the reviewer’s comment seriously and described the motivations for adopting a monitoring approach.

 Our motivations for adopting a process monitoring an evaluation approach is now highlighted under the Motivation for incorporating feedback loops section within the revised manuscript.

 This comment, together with the comment above, suggests to us that we did not do a good job in making the distinction between two things: a protocol paper about the intervention (which this paper is NOT about, and one which the reviewer seems to be focusing on) versus a protocol paper about MONITORING the intervention. We hope that the new language we have adopted in this round reflects the difference more accurately.

 To clarify, we do not state that we offer RPM4K (our digital monitoring tool) as an intervention component, rather, we use the tool to monitor the delivery of our intervention components. The innovation we offer is the incorporation of feedback loops with near-to-real-time data and we highlight and detail the contributions of our approach in the recently added Conclusion section among other sections throughout the manuscript.

 This comment, too, focuses on the intervention, rather than on its monitoring. We propose that the innovation here is in how real-time monitoring was conceptualized, implemented, and used to provide feedback to the intervention. Indeed, to the reviewer’s point, we do monitor each intervention component separately and the monitoring system is explicitly designed to be able to report back on each individual component. The reviewer makes an important point about how we know which component had what kind of effect; indeed, we have just published such a paper:

 Sedlander, E., Pant, I., Bingenheimer, J., Paltro, L., Yilma, H., Ganjoo, R., Mohanty, S., Rimal, R. (2022). How does a social norms-based intervention affect behavior change? Interim findings from a cluster randomized controlled trial in Odisha, India. BMJ Open.

 The reviewer is correct that these measures are superficial. What they offer, however, are real time quick measures. They are NOT the whole story, of course, but they minimize participant burden and provide the project a snapshot about what people like and what they dislike. Our receptivity (now renamed as acceptability) measure was limited to likes versus dislikes to reduce participation burden for our intervention participants and frontline workers alike (both groups also reported having limited digital and educational literacy). However, we also monitored receptivity (which has now been renamed acceptability) qualitatively on a regular basis. As for replicability of the intervention, our overall study protocol is pre-registered and offers clarity on the motivations, goals, aims, measures, and summative outcomes such that replicability of the intervention will be entirely possible using the details provided in the RCT protocol trial.

 Thank you for this observation and suggestion. Details regarding who collects and submits data and how these insights will inform identification of feedback loops for our tailoring variables directly related to achieving the aims of the RANI project are now detailed under the data collection section with specific sub-headings for each process evaluation measure and the identification and incorporation of feedback loops. Involvement of key actors is also outlined within these section and sub-sections. Instruments used to collect the data as well as other clarifying tools and processes are also included in the Extended data section. Finally, identifying relevant research questions, aims, and the entire evaluation framework was a collaborative effort involving local stakeholders, project team members from Odisha, and from George Washington University therefore we stand by our research questions and aims in their current form. This process is detailed in the section Process evaluations aims and questions section. The M&E framework included within the Extended Data or Supplementary Materials section brings together all of these details under one table for readers and reviewers who are curious to delve deeper into these details unavailable in the main manuscript.

 Since this is a process evaluation and monitoring protocol that aims to innovate intervention delivery in near-to-real-time with the identification and integration of feedback loops, there isn’t a theory of change we’re able to include within this protocol. The entire premise of this process evaluation approach is to “build the ship as it was sailing .” We provide our rationale and existing calls to action to adopt such a method by leading experts in the field in the Use of Feedback Loops section.

 The three distinct aims listed in our process evaluation protocol are centered around the intervention components designed for the RANI trial following a rigorous mixed-methods formative evaluation which included in-depth interviews, focus group interviews, stakeholder input sessions, and a quantitative survey. RANI team members and stakeholders identified all three areas addressed with the process evaluation aims as integral to ensuring our trial intervention delivery can proceed seamlessly. Our protocol includes specific details regarding how the feedback loops will be identified and delivered within the Use of Feedback Loops section. Table 3 provides hypothetical examples that visualize the process described under the Identifying, documenting and integrating feedback loops section. Furthermore, the feedback loops documentation template is included in the extended data section as well. All of these details in their entirety provide a detailed picture on how to apply, identifying and integrate feedback loops into process monitoring and evaluation efforts for randomized control field trials similar to the RANI project. This process evaluation protocol is centered on the use of near-to-real-time data to foster adaptive intervention delivery by integrating feedback loops during the intervention delivery process. We should also note that, in the interest of making this manuscript readable and concise, we did not repeat details provided in-depth in the RANI RANI RCT trial protocol that is described elsewhere and cited within this manuscript. We have added brief details to the RANI project description section to address all requested detailed intervention information and we’ve encouraged our readers to reference the RANI trial protocol for any and all details typically included in the trial protocol.

# Peer review: Reviewer response for version 1

**DOI:** 10.21956/gatesopenres.14672.r31875

**Published:** 2022-04-26

**Authors:** Claudia Abreu Lopes

**Affiliations:** 1 International Institute for Global Health, United Nations University, Kuala Lumpur, Malaysia

This article is a protocol for a process evaluation with feedback loops using an innovative digital tool for an intervention (RANI project) aimed at lower rates of anemia among women of reproductive age in selected villages in India. The article is relatively well structured and the research denotes thoughtfulness and rigour. The research questions are clearly stated and the methods are adequate to answer the research questions. However, the article would benefit from improvements in four main aspects:

1. The study uses a multi-method approach that consisted of collecting information from surveys, qualitative reports and structured observations. The description of the method as mixed methods is not entirely correct - multi-method would be preferable - since the methods or data sources were not triangulated or used in sequence. As it reads, different methods/data sources were used to measure different indicators/KPIs.
2. Research aims refer to implementation research outcomes, such as fidelity. The second aim is about the acceptability of the intervention, referred to in this study as 'receptivity'. The KPI refers to satisfaction which is a related but different concept. The way the data is gathered with likes and dislikes and the way the indicator is calculated are a 'black box'. For example, it says: 'Receptivity is summarized at the village-level by algorithms embedded within RPM4K'. The authors should review the concept of receptivity (acceptability would be preferable) and explain how the data is gathered and the KPIs calculated (providing/explaining the algorithm).
3. Table 1 presents 2 process evaluation questions but there are 3 process evaluation aims. The correspondence between aims and questions is not clear. Table 2 is also confusing. There may be a third measure missing as it jumps from 2 to 4 (please check other typos in Table 2 also). Table 1 can be improved by linking aims, research questions and measures. Table 2 would complement Table 1 by linking the KPIs and ecological levels to the measures. There is also not enough information to understand how the KPIs are measured at different ecological levels. For example 'why the number of women participating in hemoglobin testing is an interpersonal indicator?' Please add more information to the description of KPIs - method, data sources and calculation/formula for indicators.
4. Please add a short conclusion.

Finally, the background sentence in the abstract needs to be improved (proliferation of digital age or proliferation of digital technologies?)

Is the study design appropriate for the research question?

Yes

Is the rationale for, and objectives of, the study clearly described?

Yes

Are sufficient details of the methods provided to allow replication by others?

Yes

Are the datasets clearly presented in a useable and accessible format?

Yes

Reviewer Expertise:

Implementation research; Digital health; Mental health; Research methods; Gender and health

I confirm that I have read this submission and believe that I have an appropriate level of expertise to confirm that it is of an acceptable scientific standard.

# Response

**Published:** 2022-09-18

**Authors:** Ichhya Pant

**Affiliations:** GWU School of Public Health, USA

We would like to start by thank you for reviewing this manuscript. To respond to your feedback, we offer the following point by point response to address the concerns raised by Reviewer #1.

 Thank you for making that distinction, which is valid. Based on that distinction, however, we do indeed triangulate information collected via qualitative and quantitative surveys on a monthly basis and well as with our mid-line survey. Thus, our description of our methodology as mixed methods is intentional. This process of information triangulation in order to identify and integrate feedback loops are detailed under the sections – 1) identifying, documenting, and implementing feedback loops 2) survey data triangulation.

 We’ve added brief text to make this more explicit for our reviewers and readers.

 The KPI formerly listed as receptivity has been renamed as acceptability to address your concerns.

 We’ve also incorporated revisions to clarify that the algorithm simply summarizes the total count of likes versus dislikes for intervention components by village. Additional File 1 includes details for how each measure is operationalized and aggregated by RPM4K. Furthermore, we operationalize and define all measures at an aggregate level within the Data Collection section as well. Last but not the least, the software code for RPM4K is also publicly available in a Github repository which we’ve shared in the Extended data section. Auditing and examining how the algorithms are set up within RPM4K is possible by examining the source code as well. Our choice to make the software available publicly is supported by our funders and speaks to our intentions to veer away from black box calculations of KPIs.

 We direct the reviewer to the two evaluation questions and sub-questions listed underneath them that address all three aims. Our evaluation framework included in the Extended Data section connects Table 1 and 2 per your suggestion. In the interest of space, word count, and formatting, we aren’t able to integrate Table 1 and 2 per your suggestion here. However, the framework included in the Supplementary Material ties together the aims, questions, indicators, measures, data sources, formulas, responsible parties, operational definitions and ecological levels.

 We have added a short conclusion section as well as reframed the background sentence in the abstract per your recommendation.
